# A picture tells a thousand words: transmission electron microscopy of the ciliary transition zone in *C. elegans*

**DOI:** 10.1186/2046-2530-1-S1-P18

**Published:** 2012-11-16

**Authors:** R Bowie, K Kida, M Leroux, O Blacque

**Affiliations:** 1 University College Dublin, Ireland; 2 Simon Fraser University, Burnaby, BC, Canada

## 

Transmission electron microscopy (TEM) is a valuable tool for elucidating cilium ultrastructure and the effects of ciliary and ciliopathy gene mutations on cilium integrity. We perform TEM on amphid channel sensory cilia in *C. elegans*, enabling us to visualise cilium compartments, namely the axoneme, the transition zone (TZ) and the transition fiber/basal body region. In worms, cilia extend from the dendritic tips of 60 sensory neurons. Here we show how TEM can be used to identify ultrastructural defects observed at the TZ of a number of ciliopathy disease gene mutants. In WT worms, the ~1 micron TZ is the most proximal part of the axoneme that extends from the far distal dendrite membrane, and is defined by a drawing together of the 9 doublet microtubules (MT) and Y-links that connect the doublet MTs with the ciliary membrane. In worms with mutations in Meckel-Gruber Syndrome (MKS) and Nephronophthisis (NPHP) gene homologues, we have observed a range of TZ abnormalities. These include TZs with Y-link defects (missing arms, reduced numbers) and TZs that fail to dock at the tip of the dendritic compartment. In most instances, TZ defects are observed only in worms where both MKS and NPHP functional modules are genetically disrupted and not in worms where only one module is abrogated. Together, our observations indicate redundant roles for multiple TZ-associated ciliopathy proteins in establishing physical connections between the TZ and the ciliary membrane.

